# Hypothermia in trauma patients arriving at an emergency department by ambulance in Johannesburg, South Africa: a prospective study

**DOI:** 10.11604/pamj.2018.31.136.13615

**Published:** 2018-10-23

**Authors:** Craig Vincent-Lambert, Cecile May Smith, Lara Nicole Goldstein

**Affiliations:** 1 Department of Emergency Medical Care, Faculty of Health Sciences, University of Johannesburg, South Africa

**Keywords:** Ambulance, trauma, hypothermia, emergency department

## Abstract

**Introduction:**

Normal body temperature is considered to be between 36 and 38°C. Temperatures that are too low may negatively affect physiological functions. In trauma cases, factors that promote the development of hypothermia include concomitant hypoxia, hypotension, decreased levels of consciousness, contact with cold surfaces, exposure to low ambient temperatures and the administration of cold fluids. Studies on emergency department related hypothermia in Africa are sparse. This study investigated instances of hypothermia in a sample of trauma cases arriving by ambulance to an emergency department in Johannesburg, South Africa.

**Methods:**

Core body temperatures of 140 trauma cases were measured upon arrival and 30 minutes later. Ambient temperatures outside the hospital, inside the ED and in the resuscitation areas were also recorded. Additional information was gathered describing the equipment available to the ambulance crews for temperature, control and rewarming.

**Results:**

Seventy-two (51%) of the cases were found to have core body temperatures less than 36°C upon arrival. Twenty-nine (21%) the cases were considered clinically hypothermic (core temperatures of less than <35°C). After 30 minutes, 79 (56%) of the participants had core body temperatures of less than 36°C and 39 (28%) remained lower than 35°C. Patients were not warming up in the ED as expected. Rather, some had become colder. The study also found that the ambient temperature in the triage area fluctuated and was recorded as less than the recommended 21°C in 95 (68%) of the cases. In addition, the majority of ambulances that transported these cases lacked appropriate equipment on board to properly facilitate temperature control and rewarming.

**Conclusion:**

Fifty-one percent of the trauma cases arriving by ambulance had core temperature <36°C. Many became even colder in the ED. Attention needs to be given to the early identification of hypothermia, the regulation of ambient temperatures inside the ED including the provision of appropriate heating and rewarming devices on ambulances.

## Introduction

Maintenance of core body temperature is important to support the physiological and biological processes of the body. Normal core body temperature is considered to be between 36-38°C [1, 2]. Temperatures that are too high (hyperthermia) or too low (hypothermia) may have a negative effect on normal physiological functioning [1, 2]. The effects of hypothermia are often exaggerated in the case of underlying illness or injury [1]. Clinically significant hypothermia is seen by many as core body temperatures of < 35°C [1]. There are several clinical and environmental risk factors that promote the development of hypothermia in trauma patients [3]. Clinical factors include: hypoxia, tachycardia, hypotension and decreased level of consciousness [4]. Environmental factors involve contact with cold surfaces, exposure to decreased ambient temperatures and the administration of cold intravenous fluids [4]. The reported incidence of hypothermia in trauma cases varies and has been cited as being between 12 and 66%. In the majority of studies the coexistence of hypothermia with trauma has been linked to poorer outcomes [4-7]. Consequently the “triad of death“ has become a recognized term referring to the combination of hypothermia, acidosis and coagulopathy which, in combination, lead to an increased mortality rates [5, 8, 9]. In the triad, hypothermia is thought to impair platelet functioning by its inhibitory effect on the enzymatic reaction rate of the clotting cascade [8, 9]. The associated coagulation is not only caused by hypothermia and acidosis but can also be related to clotting factor depletion and hemodilution [9]. Acidosis (a blood pH of < 7.35) is also linked to hypothermia and blood loss as both stimulate vasoconstriction [8, 10]. The resultant vasoconstriction, coupled with the metabolic processes from shivering, stimulates a build-up of lactic acid [1, 8, 10].

Taking the above into account it is important to keep the trauma patient warm and comfortable. As the prevention of hypothermia is arguably more important than retrospectively attempting to manage it, early identification of hypothermia and maintenance of normal body temperature should be important priorities for both Emergency Department (ED) staff and pre-hospital emergency care providers. Having said this, anecdotal evidence shows that both measurement and maintenance of core body temperature are commonly overlooked and neglected in the management of trauma patients in the South African pre-hospital environment. hypothermia cannot be properly detected and managed unless patient's temperatures are routinely and accurately measured. Unlike many pre-hospital practices, measurement of body temperature in the Emergency Department (ED) are part of a routine data collection [11, 12]. Tympanic temperature measurement has become common as it remains a reliable, non-invasive and low risk procedure [12-17]. Whilst a number of international studies exist which focus on describing instances of hypothermia in trauma cases, less literature on this phenomena existed specific to the African context. The aim of our study was therefore to investigate and describe prevalence of hypothermia in trauma cases arriving by ambulance to the Emergency Department (ED). To do this we recorded the arrival temperatures of 140 trauma patients transported to a public sector emergency department by ambulance. Subsequent measurements were taken 30 min post arrival along with data relating the equipment available to ambulance crews for temperature, control and rewarming.

## Methods

A cross-sectional, observational, descriptive design was used in this study. The design was seen to be suitable for prior to the study we did not have a pre-existing data set to mine and analyse [18-19]. The study was conducted in a large academic public hospital situated in an urban area in Johannesburg, South Africa. At the time of the study the Emergency department of the hospital was open 7 days a week, 24 hours a day. We used a purposeful, convenient sampling strategy by enrolling patients who fulfilled the inclusion criteria upon their arrived at the Emergency Department. A pragmatic approach was adopted with regard to sample size. The size of the sample was therefore predetermined and informed by the study aim, period, and predicted caseload with regard to the mode of transport (public vs private ambulance) and patient acuity/priority. The inclusion criteria was all patients who had experienced trauma and who were transported by ambulance to the participating Emergency Department. Medical cases and those arriving at the Emergency Department via public transport were excluded from the study. The final sample consisted of 140 trauma cases arriving at the Emergency Department by ambulance. This sample was divided equally between private and public ambulance cases. Each set of 70 patients was further divided into 10 Priority 1, 30 Priority 2 and 30 Priority 3 cases. Ambient temperatures outside the hospital, in the resuscitation and the triage areas as well as each patient's core temperature were recorded on their arrival at the Emergency Department. The patient temperatures were again recoded 30 min post their arrival. Data was collected in the months June and July 2016; these months are considered the colder months in the year in Johannesburg. An infrared tympanic thermometer (Model AC322, Chinasia Products Ltd, Hong Kong, China) was used to measure the patient's temperatures. The infrared tympanic thermometer used in this study was newly purchased and calibrated. An ambient thermometer (WBGT heat stress monitor, model T-32/34, Quest laboratory instruments) was placed outside of the Emergency Department where the ambulances arrived. A second ambient thermometer recorded the temperature in the triage area (Wireless Weather Station, Vantage Vue^^®^^, Davis^^®^^, USA and Canada) and a third in the resuscitation room. A specifically designed template was used to record data relating to the age, gender and nature of injuries, including the 3 measured temperatures, mode of transport, equipment available to the ambulance crews for temperature control along with evidence of any efforts at warming or maintaining core body temperature. The raw data was captured onto excel spreadsheets. Thereafter data was summarised and analysed descriptively.

**Ethical considerations**: Ethical approval for this study was obtained from the University of Johannesburg's Faculty of Health Science Research Ethics Committee (REC01-234-2015) as well the Chief Executive Officer of the participating hospital. Due the nature of the study there were no anticipated risks for the participants. The researchers introduced themselves to prospective participants and provided them with an explanation of the study and its purpose upon arrival in the Emergency Department. Those who agreed to participate signed the informed consent forms.. Anonymity in this study was guaranteed in that we did not record the patient's names, addresses or other identifying data that could in any way link them to the data any stage either during or after the study. The participating hospital is also not named in the write-up or presentation of results.

## Results

Table 1 summarises the temperatures for all 140 cases on arrival and after 30 minutes including the ambient temperatures in the triage area and resuscitation room during this time. Table 2 summarises the above data by patient priority and Table 3 shows the differences between patients brought in by public and private services. An additional set of data was gathered that related to the type of equipment that was available on the ambulances for purposes of warming and temperature control and whether or not any of the listed devices had been used in the treatment and transport of the 140 cases. A summary of this is presented in Table 4. Of the 140 cases 72/140 (51.4%) were found to have lowered core body temperatures less than 36°C and 29/140 (20.7%) cases were hypothermic (<35°C) upon arrival. 30 minutes post arrival 79/140 (56.4%) of the cases temperatures were still less than 36°C and 39/140 (27.9%) of the cases remained <35°C.

**Table 1:** Temperatures measured (°C)

	Arrival	After 30 min	Outside temperature	Triage area	Resuscitation area
Minimum	32.3	32.7	4.0	16.6	18.3
Quintile 1	35.1	34.8	11.8	18.9	21.8
Median	35.9	35.8	14.7	19.5	24
Quintile 3	36.3	36.2	19	21.6	25
Maximum	37.3	37.6	26.9	22.7	29.5
Mean	35.6	35.5	15.7	19.9	23.5
Standard deviation	1.0	1.0	5.1	1.5	2.3
Range	5.0	4.9	22.9	6.1	11.2

**Table 2:** Priority one; priority two and priority three patient’s temperatures upon arrival

	Priority one*(°c)	Priority two**(°c)	Priority three**(°c)
Minimum	32.3	33.2	33.2
Quintile 1	35.0	34.8	35.6
Median	35.7	35.7	36.1
Quintile 3	36.2	36.2	36.5
Maximum	37.2	37	37.3
Mean	35.3	35.5	35.9
Standard deviation	1.3	0.9	0.9
Range	4.9	3.8	4.1

**Table 3:** Private vs public ambulance cases

	Private	Public
Minimum	33.2	32.3
Quintile 1	35.6	34.7
Median	36.1	35.8
Quintile 3	36.4	36.3
Maximum	37.2	37.3
Mean	35.9	35.4
Standard deviation	0.8	1.1
Range	4	5

**Table 4:** Equipment for temperature control available on ambulances

Item	Item used n=140	Private ambulances n=70	Public ambulances Ambulance n = 70
Sheet	45/140 (32.1%)	29/70 (41.4%)	16/70 (22.9%)
Blanket	54/140 (38.6%)	31/70 (44.3%)	23/70 (32.9%)
Space blanket	8/140 (5.7%)	3/70 (4.3%)	5/70 (7.1%)
Warm fluid	1/140 (0.7%)	1/70 (1.4%)	0/70 (0%)
Other warming device	26/140 (18.6%)	21/70 (30%)	5/70 (7.1%)

**For priority one patients**: 13/20 (65%) of priority one patients arriving with temperatures of less than 36°C; 5/20 (25%) of PRIORITY ONE patients had temperatures of less than 35°C and; 4/20 (20%) had admission temperatures of less than 34°C. The coldest PRIORITY ONE case was a drowning victim who had an admission temperature of only 32.3°C.

**For priority two patients**: 36/60 (60%) of priority two patients had an admission temperature below 36°C; 16/60 (26.7%) of PRIORITY TWO patients were hypothermic upon admission.

**For priority three patients**: 23/60 (38.3%) of priority three patients were found to have an admission temperature below 36°C; 8/60 (10%) of PRIORITY THREE patients were hypothermic upon arrival at the emergency department. The above results show that patients coming in by private ambulance were generally warmer that those that came via public sector ambulance. However, both groups had a number of admissions that were below what is considered normal with 32/70 (45.7%) of private cases being less than 36°C compared to 40/70 (57.7%) for public patients and 5/70 (7.1%) of the private patients being clinically hypothermic (<35°C) upon admission compared to 24/70 (34.3%) public patients.

## Discussion

In both the pre-hospital and Emergency Department settings it is important to control and maintain the core body temperature of patients. hypothermia is an independent risk factor in increasing morbidity and mortality in trauma cases. As shown in Table 1 just over half of the patients 72/140 (51.4%), in our study were found to have core body temperatures of less than 36°C upon arrival. A further 29/140 (20.7%) were clinically “hypothermic” (Temp < 35°C) upon admission. Possible reasons for this could be that managing the patient's temperatures may not have been seen as a priority during prehospital care with rewarming being delayed in favour of rapid transport. Alternatively, the data in Table 4 indicates that many of the ambulance crews simply did not have the resources available to properly manage the patient's temperatures. Another reason for our results could be that the patients had been lying in a cold prehospital environment for an extended period prior to the arrival of the ambulance. The patient's temperatures were reassessed 30 minutes post their arrival at the Emergency Department. Here we found that in 79/140 (56,4%) of the cases the patients came in “cold” with temperatures less than 36°C and 39/140 (27.9%) of patients remained hypothermic after 30 minutes. Strangely, we found that patients seemed to be getting colder in the Emergency Department with the percentage of those whose core body temperature were less than 36°C increased from 51.4% upon arrival to 56.4% 30 minutes' post arrival. This was an unexpected finding as we would have expected patients to warm up once inside the hospital. Whilst the exact causes for this are unknown, there are a number of factors that may have contributed to this finding. Firstly, it was noted that, once the ambulance crews handed the patients over to the Emergency Department staff they took back all of their equipment including their sheets and blankets. Thereafter the majority of the patients in the triage area were not seen to be receiving a warmed replacement blanket. Secondly, lower than desirable ambient temperatures inside the Emergency Department may also have played role. It is recommended that emergency departments should maintain an inside ambient temperature of between 21 and 24°C [4]. The ambient temperature in the triage area of the participating facility was measured at less than the recommended 21°C in 95/140 (67.9%) of the cases. This may be due to the design of the facility where the main entrance to the Emergency Department opens into the passage that communicated directly with the triage and waiting areas. This would make it difficult to control the inside ambient temperature in triage and waiting areas on cold days when the entrance doors were left open.

Our results also appear to show a possible correlation between the severity of the patient's injuries and their core body temperatures with core body temperatures of priority one patients being lower than that in priority two and priority three cases. this finding is supported by literature that indicates that serious injuries are more commonly associated with accidental hypothermia [20]. The reason for these results could also be that in the case of critical patients ambulance crews may have been focused on the performance of life-saving interventions associated with airway management, breathing and circulation support paying less attention to measuring, and managing the patients' temperature. Table 3 illustrated the incidence of hypothermia in private versus public ambulance cases. The incidence of hypothermia in the public sector was higher than in the private sector. Of the private ambulances cases 32/70 (45.7%) had core body temperatures less than 36°C and 5/70 (7.1%) were clinically hypothermic (<35°C). This is less compared to the 40/70 (57.1%) of the public sector patients whose temperatures were below normal and the 24/70(34.3%) that were clinically hypothermic (<35°C). The fact that only around 18% of South Africans are medically insured leaves the state sector with a huge burden [21]. Public sector ambulance services remain poorly equipped and regulated with many lacking basic equipment, ranging from blankets and fluid warmers to defibrillators [21]. Many of the arriving public sector ambulances were noted either to not have heaters in the rear (patient compartment), or the heaters were broken. Thus, crews were unable to effectively increase the ambient temperature inside the patient compartment of the ambulance. This is unfortunate, as passive rewarming has been shown to be a very effective method to increase the patient's temperature and reduce cold discomfort during transport [4]. Clinically significant hypothermia however requires a more aggressive approach involving active rewarding. In this study, we noted only two patients of the 140 received warmed intravenous fluids. Thus, virtually all the trauma cases who were cannulated received cold fluids. This is hugely problematic for the administration of cold intravenous fluid in trauma patients increases the probability of developing hypothermia and may be another possible reason as to why the patients in this study were not warming up in the Emergency Department [4].

## Conclusion

Hypothermia in the context of trauma is clinically undesirable and has been associated with poorer outcomes [22]. The results of this study showed a number of trauma cases arriving by ambulance to the Emergency Department were already “cold”. Certain of these did not warm up as quickly as expected and in fact, some become colder lying in the triage area. There are several clinical and environmental risk factors that promote the development of hypothermia in trauma patients [3]. Clinically significant hypothermia is seen by many as core body temperature of < 35°C [1]. Reduced core body temperatures below 34°C have been linked to a significant increase in posttraumatic complications. Core body temperatures of less than 32°C have been associated with increased mortality rates [9, 22]. In this study 29/140 (20.7%) of the cases were clinically “hypothermic” (< 35°C) upon admission and a further 13/140 (10%) had core body temperatures less than 34°C. Potential reasons for these findings are discussed in this report and include; a possible lack of attention and awareness of the importance of temperature control in trauma cases, poor regulation of ambient temperatures inside the Emergency Department, administration of cold intravenous fluids by ambulance crews and a lack of appropriate rewarming equipment on ambulances. We acknowledge that this study was purely descriptive and exploratory in nature. Our study only looked at data from one public sector urban ED and we also concede these results may differ between Emergency Departments and ambulance services in other regions of the country and continent. Our results may also have been different if data was only gathered during times when the ambient outside temperatures were low i.e. night shifts. However, in our case we gathered data focusing on achieving the total desired sample size of 140 rather than purposefully looking to ensure an equal number of day and night shifts. Although we mentioned in our report that tympanic thermometers are commonly used and are seen as a reliable measurement method for assessing patient's temperatures they do have limitations and are generally considered less accurate than indwelling devices. Finally, the data was gathered during the months of June and July, which as mentioned fall during winter and for this reason the data may differ should the study be conducted during other times of the year. Future studies may be considering that look into the attitudes, behaviours and understanding of pre-hospital emergency care workers and triage nurses about their identification and management of “cold” trauma cases.

### What is known about this topic

- Hypothermia in the context of trauma is clinically undesirable and has been associated with poorer outcomes;
- Reduced core body temperatures below 34°C have been linked to a significant increase in posttraumatic complications.

### What this study adds

- Swapping of blankets and poor regulation of ambient temperature control inside the Emergency Department appear to have a significant effect on core body temperatures of trauma patients during admission;
- Administration of cold intravenous fluids by ambulance crews and a lack of appropriate rewarming equipment and cabin temperature control in ambulances contribute to instances of hypothermia seen on admission;
- Ambulance crews and in hospital staff need to be more aggressive in their detection and management of trauma cases with concomitant hypothermia.

## Competing interests

The authors declare no competing interests.

## Authors’ contributions

Craig Vincent-Lambert supervised the study and wrote this article. Cecile May Smith gathered data and completed the initial report upon which this article is based. Lara Nicole Goldstein assisted in data gathering and interpretation of results. All authors approved the final version submitted for publication.
